# Designing Prosthetic Hands With Embodied Intelligence: The KIT Prosthetic Hands

**DOI:** 10.3389/fnbot.2022.815716

**Published:** 2022-03-10

**Authors:** Pascal Weiner, Julia Starke, Samuel Rader, Felix Hundhausen, Tamim Asfour

**Affiliations:** High Performance Humanoid Technologies Lab, Department of Informatics, Institute for Anthropomatics and Robotics, Karlsruhe Institute of Technology (KIT), Karlsruhe, Germany

**Keywords:** humanoid hands, prosthetic hand, grasping, embedded systems, underactuation, embedded sensing, sensor-based grasping

## Abstract

Hand prostheses should provide functional replacements of lost hands. Yet current prosthetic hands often are not intuitive to control and easy to use by amputees. Commercially available prostheses are usually controlled based on EMG signals triggered by the user to perform grasping tasks. Such EMG-based control requires long training and depends heavily on the robustness of the EMG signals. Our goal is to develop prosthetic hands with semi-autonomous grasping abilities that lead to more intuitive control by the user. In this paper, we present the development of prosthetic hands that enable such abilities as first results toward this goal. The developed prostheses provide intelligent mechatronics including adaptive actuation, multi-modal sensing and on-board computing resources to enable autonomous and intuitive control. The hands are scalable in size and based on an underactuated mechanism which allows the adaptation of grasps to the shape of arbitrary objects. They integrate a multi-modal sensor system including a camera and in the newest version a distance sensor and IMU. A resource-aware embedded system for in-hand processing of sensory data and control is included in the palm of each hand. We describe the design of the new version of the hands, the female hand prosthesis with a weight of 377 g, a grasping force of 40.5 N and closing time of 0.73 s. We evaluate the mechatronics of the hand, its grasping abilities based on the YCB Gripper Assessment Protocol as well as a task-oriented protocol for assessing the hand performance in activities of daily living. Further, we exemplarily show the suitability of the multi-modal sensor system for sensory-based, semi-autonomous grasping in daily life activities. The evaluation demonstrates the merit of the hand concept, its sensor and in-hand computing systems.

## 1. Introduction

Hand prostheses allow amputees to regain autonomy and abilities in their daily life. Recent advances in prosthetic hand development have led to sophisticated multiarticulated devices. However, the rejection rate of myoelectric prostheses is very high with 18–23% and another 30% only use their myoelectric prosthesis as a passive device according to Biddiss and Chau (2007) and Østlie et al. (2012). One cause for this problem arises from limitations in terms of intuitiveness-of-use and a high level of user control effort to execute grasping tasks. These limitations can be relaxed by the integration of intelligent hardware and software. Underactuated mechanisms have proven to be a very promising way to design robot hands with a small number of active degrees of freedom (DoF) as shown for example by Fukaya et al. (2000), Belter and Dollar (2013), and Catalano et al. (2014) amongst others. Such hands are able to adapt to the shape of objects to reliably execute grasps while exploiting the physical interaction with the object. In addition, intelligent control strategies significantly reduce the cognitive burden on the user by taking information about the environment and user intention into account to autonomously select suitable grasps while keeping the user in the loop. Such semi-autonomous control strategies are an emerging research topic with several recent developments e.g., by Došen et al. (2010), Markovic et al. (2015), and Ghazaei et al. (2017) amongst others. However, semi-autonomous control requires profound knowledge about the environmental situation and the user intention which must be acquired by an appropriate sensor system and sufficient computing resources to extract such knowledge from sensor data. In particular, visual perception is key for scene understanding which is needed to recognize and segment objects to be grasped.

**Figure 1:**
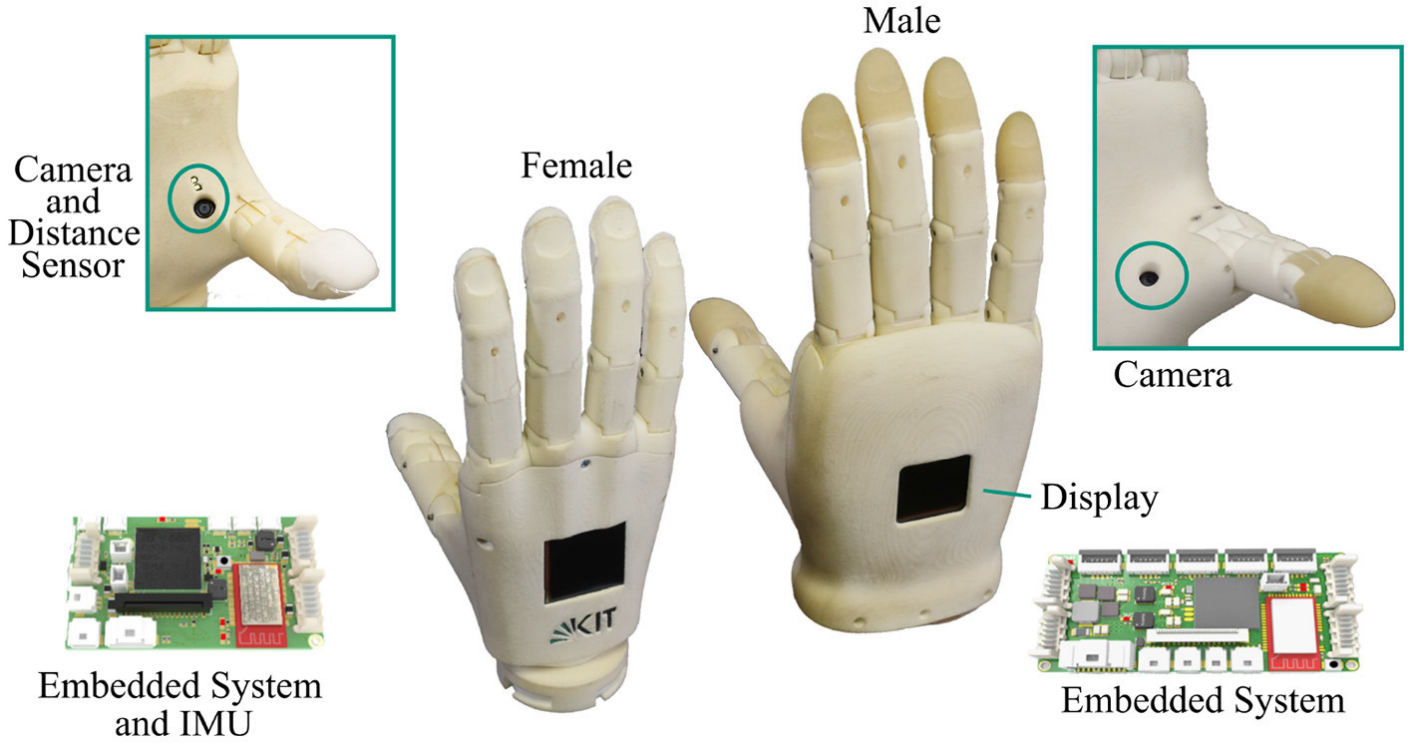
The KIT Prosthetic Hands; female (left) and male (right) intelligent hand prostheses designed for semi-autonomous grasp control. Each hand has two DC motors actuating 10 DoF via an underactuated mechanism. Each hand is equipped with a camera in the palm, IMU and a distance sensors (female version) as well as an integrated embedded system for in-hand sensor data processing and control.

In this paper, we present our recent work on the development of highly integrated prosthetic hands that are equipped with on-board sensors and computing power to support the realization of semi-autonomous grasping. The hands, as depicted in Figure 1, are driven by two DC motors, one motor for the thumb and one for the fingers, with a total of 10 DoF. Specifically, we describe the new version of our prosthetic hands, the female version, which is based on our previous work regarding the design of prosthetic hands in Weiner et al. (2018) and in-hand visual data processing for object detection by Hundhausen et al. (2019). The female prosthesis extends our previous work on the male hand in terms of sensing and visual perception capabilities, underactuated mechanism and shows the scalability of our design to different hand dimensions.

The paper is organized as follows: Section State of the Art provides an overview over the state of the art in prosthetic hand design. In Section Key Requirements the key requirements governing the development of our hand prostheses are explained. The mechanical design as well as the sensors and embedded system are detailed in Section Design and Mechatronics. Section Evaluation describes experimental results regarding main characteristics and real-world grasping studies. The paper concludes with a summary and discussion in Section Evaluation.

## 2. State of the Art

Throughout the last two decades the development of artificial humanoid hands has made considerable progress regarding mechatronics and control strategies of such hands. A comprehensive list with a broad overview of hand development is given by Piazza et al. (2019), including both prostheses and robotic hands. Table 1 gives an overview of recent developments in myoelectric hand prostheses with a special focus on built-in intelligent capabilities that are needed for semi-autonomous control.

**Table 1:** Overview of commercial and research prostheses.

**Prosthesis**	**Actuation**				**Sensors**					**Mechanical characteristics**		
	**DoF**	**DoA**	**Adap. Un.^a^**	**Emb. Sys.^b^**	**Position^c^**	**Force**	**Orientation**	**Vision**	**Distance**	**Size^d^**	**Weight^e^**	**Force^f^**
SensorHand (SensorHand, 2020)	2	1	◯	●	n.a.	●	◯	◯	◯	178–210 l	460	100 PG
iLimb pulse (Belter and Dollar, 2013)	11	5	◯	●	n.a.	◯	◯	◯	◯	180–182 l x 75–80 w x 35–45 h	460–465	6.2–11.8 FF
Bebionic (Belter and Dollar, 2013)	11	5	◯	●	n.a.	◯	◯	◯	◯	190–200 l x 84–92 w x 50 h	495–539	12.3–16.1 FF
Michelangelo (Belter and Dollar, 2013)	6	2	◯	●	n.a.	n.a.	◯	◯	◯	180 l	420	70 P
Vincent hand (Belter and Dollar, 2013)	11	6	◯	●	n.a.	n.a.	◯	◯	◯	145–180 l x 65–85 w	386 (XS)	4.8–8.4 FF
Taska Hand (Taska, 2020)	10	6	◯	●	n.a.	n.a.	◯	◯	◯	179–181 l x 81–88 w	556–671	6.7–22 FF
MANUS-Hand (Pons et al., 2004)	10	3	◯	●	●	●	◯	◯	◯	1.2*50th percentile male	1200^g^	60 PG
HIT/DLR Prosthetic hand (Huang et al., 2006)	13	3	S	●	●	●	◯	◯	◯	n.a.	n.a.	n.a.
CyberHand (Carrozza et al., 2006)	16	6	◯	◯	●	●	◯	◯	◯	n.a.	360	70 PG
SmartHand (Cipriani et al., 2011	16	4	S	●	●	●	◯	◯	◯	50th percentile male	520	16–36 PG
Vanderbilt (Wiste et al., 2011)	16	4	S	◯	◖	◯	◯	◯	◯	n.a.	320	10–34 FF
UT Hand I (Peerdeman et al., 2014)	15	3	W	◯	●	●	◯	◯	◯	185 l x 82 x w x 26 h	n.a.	n.a.
Vanderbilt 2 (Bennett et al., 2015)	9	4	S	●	◖	◯	◯	◯	◯	200 l x 89 w	546	15–30 FF
SoftHand Pro-D (Piazza et al., 2016)	19	1	T	●	◯	◯	◯	◯	◯	235 l x 230 w x 40 h	n.a.	20 PG
SSSA-MyHand (Controzzi et al., 2017)	10	3	◯	●	●	◯	◯	◯	◯	200 l x 84 w x 56 h	478	9.4–14.6 FF
Jeong et al., 2017	11	6	◯	◯	◯	●	◯	◯	◯	Average Male	380	15.7–48.2 FF
SCCA Hand (Wiste and Goldfarb, 2017)	11	5	S	◯	◖	◯	◯	◯	◯	n.a.	437	146 PG
SoftBionic hand (Tavakoli et al., 2017)	10	2	T	●	◖	●	◯	◯	●	200 l x 91 w x 40 h	285	n.a.
Zhang et al. (2018)	11	6	T	●	◖	●	◯	◯	◯	171 l x 80.2 w x 27.4 h	450	8-12 FF
PRISMA Hand II (Liu et al., 2019)	19	3	S	◯	◖	●	◯	◯	◯	210 l x 80 w	n.a.	n.a.
Galileo hand (Fajardo et al., 2020)	15	6	◯	●	◖	◯	●	◯	◯	162 l x 69.6 w x 25 h	350	50 PG
KIT Prosthetic hand male (Weiner et al., 2018)	10	2	W	●	◖	◯	◯	●	◯	232^h^ l x 87 w x 35 h	768	6.2-8.2 FF, 24.2 PG
KIT Prosthetic hand female	10	2	T	●	◖	◯	●	●	●	194^h^ l x 77 w x 28 h	377	9.0-12.3 FF, 40.5 PG

a *Adaptive underactuation of multiple fingers, S for spring-based mechanism, T for tendon-based mechanism, and W for whippletree-based mechanism*;

b *Embedded system integrated*;

c *●in case of joint angle encoders and ◖for motor relative encoders*;

d *Dimensions in mm, l: length, w: width, h: height*;

e *Measured weight in Gramm*;

f *Measured force in Newton, PG: Power Grasp, FF: Finger Forces, P: Pinch*;

g *Including wrist and socket*;

h *Including hand adapter; ◯: not included, n.a.: unknown*.

As can be seen from the degrees of freedom (DoF) and degrees of actuation (DoA), all prosthetic hands make use of underactuation to reduce the number of motors and overall control complexity. Most research prostheses address the question of how to realize adaptive hand behavior. In the SmartHand by Cipriani et al. (2011), one motor drives three fingers by an adaptive mechanism using series elastic elements to integrate compliance into the design while being not back-drivable due to a spindle drive. The Southampton Hand by Kyberd et al. (2001) actuates middle, ring and little finger with a lever-linkage mechanism allowing adaptive finger closing. The SoftHand Pro-D by Piazza et al. (2016) and the Hannes Hand by Laffranchi et al. (2020) utilize a single motor to drive all fingers via tendons.

The trade-off between size, weight and force is an important consideration for both commercial and research prostheses. Table 1 shows that while the weight of most research prostheses is well in the range of the human hand weight, both size and grasping forces vary considerably.

Several sensors as well as an embedded system are commonly used in research prostheses. Position sensing is implemented by almost all prostheses either by means of motor relative encoders or joint angle encoders. Joint angle encoders have the advantage that the kinematic state of the prosthesis is completely known, while motor relative encoders in underactuated hands often only allow for an estimation of the state of the hand. Force sensing is either implemented by integrating tactile sensors into the fingertips, load cells inside the finger structure or in series with the tendons. Zhao et al. (2016) recently also introduced flexible tactile sensors to prosthetic hands, covering the whole finger surface. Further, several grasp force controllers have been proposed, such as in Pons et al. (2004), Carrozza et al. (2006), Huang et al. (2006), and Tavakoli et al. (2017) among others. Other sensor modalities such as cameras, distance sensors or IMUs are not yet readily available in hand prostheses, as can be seen in Table 1. Several prostheses integrate an embedded system based on one or more microcontrollers, which is mostly used for low-level motor control.

## 3. Key Requirements

To provide support for the user performing diverse activities of daily living (ADL), as for example food preparation, housekeeping or tool use amongst others, a prosthesis has to be reliable and versatile in terms of its grasping capabilities, i.e., it should be able to successfully perform a wide variety of ADLs (Matheus and Dollar, 2010). The user expects their prosthesis to be effortless and intuitive despite the inherent complexity of the mechatronics and control (Cordella et al., 2016). The pivotal point of our hand development is therefore to endow prosthetic hands with intelligent grasping capabilities to support intuitiveness-of-use and to reduce the cognitive burden of the user. In this work, we strive for intelligent hand mechatronics, that provide the sensor information and capabilities to render intuitive, partially autonomous grasp control possible. In the following, we discuss the key requirements that should be taken into account in the context of the development of such prosthetic hands. These concern the simplicity of mechanical design, the ability to perceive and interpret the current scene, the computing system needed for sensor data processing and control as well as requirements regarding size, weight and appearance of the hand. Underactuated mechanical designs have shown how grasping behavior can be achieved by intelligent hand and finger mechanisms that are able to autonomously self-adapt the hand morphology to the object shape, see Pfeifer and Gómez (2009) and Carrozza et al. (2006). This allows the realization of basic grasping by exploiting the interaction of the hand with the object while using simple and often none precise control.

While such self-adaption of the hand reduces the control complexity for closing the hand, it does not simplify other parts of a grasping action for the prosthetic hand user. This includes the selection of a grasp type, hand preshape and hand orientation, which depend on the object to be grasped and on task-specific constraints. Thus, an intuitive-to-use prosthetic hand should be able to autonomously determine suitable grasps, hand preshapes and orientations based on the available object information and the user intention. To keep the human in the loop, the execution of the different parts of a grasping action should always be supervised by the user leading to semi-autonomous grasping behavior. Different semi-autonomous control schemes have been proposed in literature and have proven to reduce the cognitive burden for the user (Došen et al., 2010; Markovic et al., 2015; Ghazaei et al., 2017).

To achieve such semi-autonomous grasping behavior, a multi-modal sensor system is needed to perceive the scene, extract important object information as well as to capture user's state and intention. Visual perception plays a key role for scene understanding, in particular for object detection that is needed to generate suitable grasps. Thus, vision systems have been a central part of semi-autonomous grasping setups, with cameras attached to the human body or the environment to provide the necessary information. In our work, we integrate a camera, an IMU and a distance sensor in the prosthesis to provide a fully integrated system enabling semi-autonomous grasping. In addition, according to Cordella et al. (2016), providing feedback to the user about the state of their prosthetic hand is important and should be considered. For processing and interpretation of multi-modal sensory data, appropriate computing resources are needed that can be integrated in the hand while taking into account space limitations and energy consumption. In addition, resource-aware image processing and machine learning methods are needed.

Finally, the hand needs to comply with the general design requirements for prosthetic hands in terms of size, weight, grasp force, speed and appearance (Pylatiuk et al., 2007; Wijk and Carlsson, 2015; Cordella et al., 2016; Schweitzer et al., 2018). Thus, the design of the prosthetic hand should take into account the scalability in size to fit a large portion of the population. To show the feasibility of integrating the intelligent functions described above within the severe space limitations of prostheses, we design a hand with the size of a 50^th^ percentile female hand according to the German standard specification (DIN 33402-2). According to the literature, the weight of the prosthetic hand should not exceed 400 g to match the weight of a human hand (Kaye and Konz, 1986). Further, the grasping force and closing speed of the hand should be comparable to commercial hands, as reported in Belter and Dollar (2013).

## 4. Design and Mechatronics

The KIT Prosthetic Handis an underactuated myoelectric prosthetic hand driven by two motors and controlled via muscle signals extracted by *electromyography* (EMG). In this section we present the mechanical and electrical design of the female prosthesis offering mechanical grasp support via underactuation and providing a platform for intelligent and context-aware control algorithms. The advances in design are shown in comparison to the male prosthesis described in Weiner et al. (2018).

### 4.1. Actuation and Adaptive Mechanism

**Figure 2:**
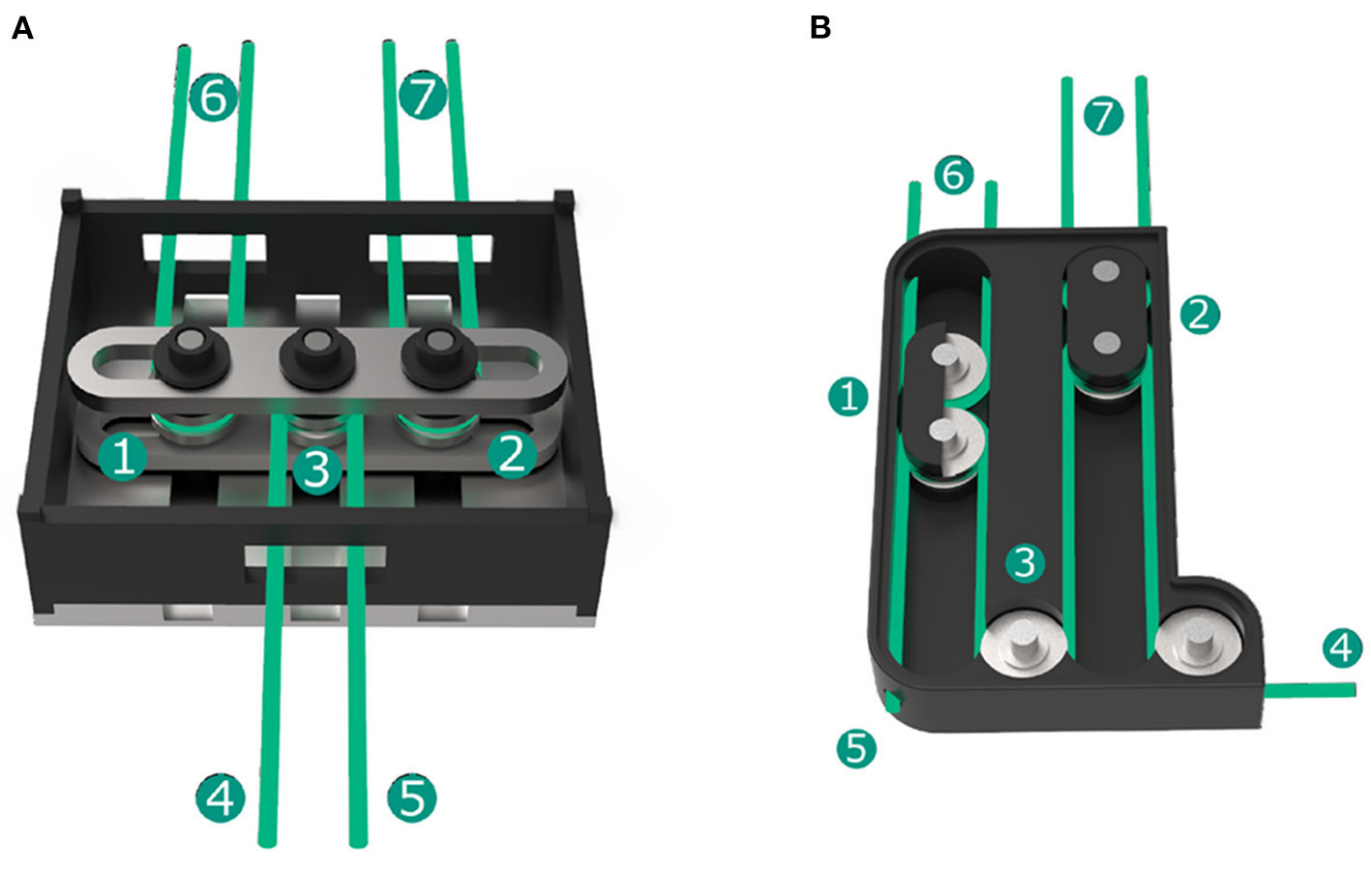
Underactuated force distributing mechanism for the fingers; the mechanism in the male hand connects two fingers by a single tendon and the pairs of fingers by a lever **(A)**; the mechanism in the female hand actuates pairs of fingers by free floating sliders interconnected by the motor tendon **(B)**.

The design of the prosthesis incorporates two DC motors (2224U012SR, Faulhaber) that are equipped with relative encoders (IEH2-512, Faulhaber) and a planetary gear (Series 20/1R, Faulhaber) with 23:1 transmission. The first motor drives the thumb flexion. All four fingers are actuated together by the second motor via an underactuated mechanism. Both versions of the mechanism in the male and female hand are depicted in Figure 2.

For the male hand, we presented the mechanism in Figure 2 consisting of a rocker that is centrally pulled by a tendon on pulley ③ connected to the motor at ④. The tendons ⑥ and ⑦ connecting two fingers each are fixed on either side of the lever bar and rotate around the floating pulleys ① and ②. As long as all of the fingers can close freely, all finger tendons are pulled equally causing finger flexion. If one finger is blocked by an object, the tendon turns around its pulley, thereby further closing the second finger connected to the same tendon. If both fingers connected to a tendon cannot close any further, the lever of the mechanism rotates and allows the other two fingers to continue closing. This mechanism design provides the prosthesis with the ability to wrap around arbitrarily shaped objects without the need of complex control input.

In the female hand, the mechanism is further improved regarding the required input force, sizing and friction. The lever is replaced by two separate sliders ① and ② consisting of two connected pulleys. The sliders are free floating and move along their individual guides. The tendon coming from the motor at ④ is led around one pulley of slider ②, a fixed guiding pulley ③ and to slider ①, before it is fixed at the housing at ⑤. The tendons ⑥ and ⑦ connecting two fingers each are led around the second pulley of one slider each. By pulling the motor tendon, the force is still equally distributed to all four fingers by similarly actuating both sliders. The distribution between two fingers remains the same as in the first hand version while the lever is replaced by the two sliders distributing force between the individual pairs of fingers.

Apart from the reduced dimensions of the mechanism, the additional redirection of the tendon between both sliders results in a force transmission ratio of 2:1, thereby doubling the finger force compared to the tendon force on the motor pulley. Together with a decrease of the diameter of the motor pulley from 16 to 8 mm, which corresponds to an additional transmission ratio of 2:1, this allows the reduction of the transmission gear of the motor by factor four from 86:1 in the male hand to 23:1 in the female hand. Therefore, the gear needs one reduction stage less, hence making the gear shorter and lighter while also increasing transmission efficiency.

As the sliders are held in constant tension between motor and finger tendon, they are free-floating and thereby cause no friction against the mechanism walls. All pulleys are supported by ball bearings. This further reduces the friction within the mechanism, thereby increasing the resulting finger force. The design with individual sliders makes the mechanism suitable to be used with other finger designs. This has been shown in the development of the KIT Finger-Vision Soft Hand described by Hundhausen et al. (2020) in which three fingers are driven with an adapted version of this mechanism.

### 4.2. Mechanical Design

**Figure 3:**
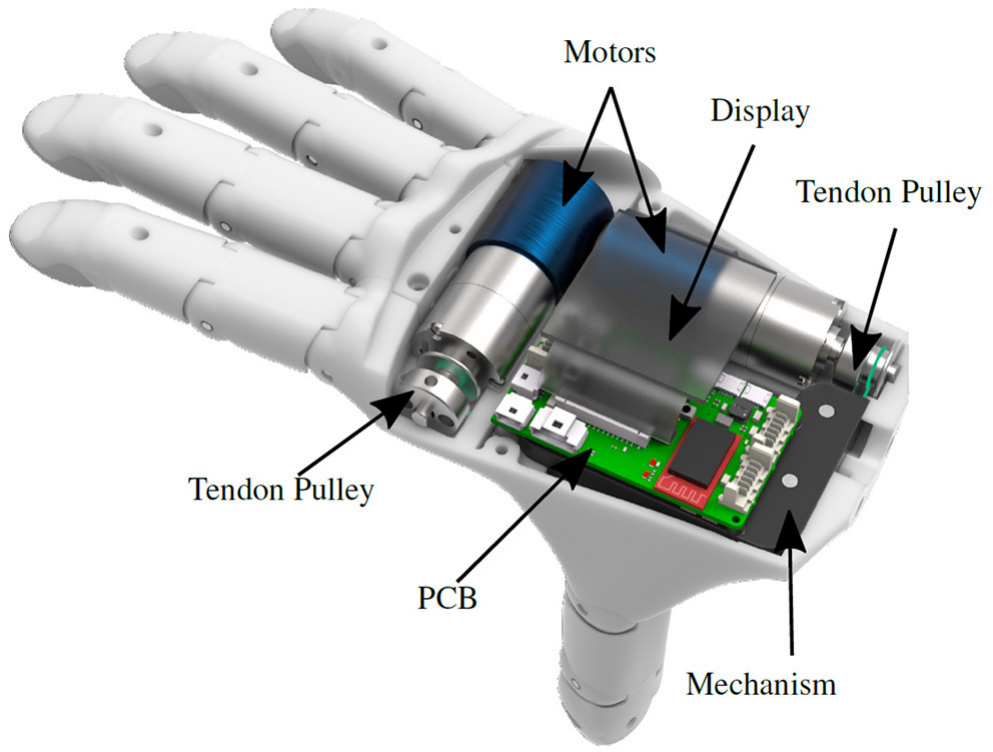
The female prosthesis with motors, mechanism and PCB integrated into the palm. Camera and distance sensor are mounted below the mechanism. The mechanism in black is mounted below the PCB. The display is fixed on top of the PCB in the dorsal housing. The display is rendered semi-transparent to make the components underneath visible.

The mechanism and motors are placed within the palm of the hand together with the sensors and the embedded system, as shown in Figure 3. The male and female prostheses have the size of a 50^th^ percentile male and female hand, respectively, according to the German standard specification (DIN 33402-2). Individual finger segment lengths are based on the human hand length study by Vergara et al. (2016). The dimensions of both prosthetic hands are listed in Table 2.

**Table 2:** Dimensions of the KIT prosthetic hands.

**Hand part**		**Male (mm)**	**Female (mm)**
Palm	Length	111	100
	Width	87	77
	Depth	30	26
Thumb	Proximal phalanx	37.0	32.7
	Distal phalanx	37.7	33.2
Index finger	Proximal phalanx	29.9	27.0
	Intermediate phalanx	28.0	26.4
	Distal phalanx	27.1	25.5
Middle finger	Proximal phalanx	33.6	30.3
	Intermediate phalanx	32.3	30.4
	Distal phalanx	28	26.3
Ring finger	Proximal phalanx	30.1	26.9
	Intermediate phalanx	31.3	29.3
	Distal phalanx	28.6	26.8
Little finger	Proximal phalanx	22.8	20.5
	Intermediate phalanx	23.9	22.6
	Distal phalanx	27.3	25.7

Despite a reduction of the integration space by 30.9 % compared to the male hand, all hardware components including the two motors, the underactuated mechanism, sensors and the embedded system are integrated into the palm of the female prosthesis. The fingers are designed based on a CAD model, which allows scaling of the hand according to the size of the user's able hand. To support a lightweight design, the housing, finger phalanges and mechanism sliders are 3D-printed using selective laser sintering from polyamide (PA2200), a robust, yet flexible plastic.

The fingers are actuated by 0.4 mm Dyneema tendons. Each finger comprises actuated flexion in the *metacarpophalangeal joint* (MC joint) and the *proximal interphalangeal* joint (PI joint). The *distal interphalangeal joint* (DI joint) is fixed at an angle of 20°. The resulting 10 joints are equipped with ball bearings and the tendon is routed through Teflon tubes (PTFE) to minimize friction. Torsion springs are included in the finger joints and support the passive extension of the fingers. A higher pretension of the springs in the PI joints leads to a higher closing speed of the MC joints compared to the PI joints. This results in a human-like spiral fingertip closing trajectory, as shown by Kamper et al. (2003).

The fingertips are equipped with high friction finger pads to enhance the friction with the grasped object and thereby lower the required force to perform a stable grasp. The pads cover the palmar side of the medial and distal phalanges and envelop the tip as well as radial and ulnar side of the distal phalanx. They are cast from silicone and glued to the fingertip housing structure.

### 4.3. Embedded Sensor System

Both male and female prosthetic hands contain a multi-modal sensor system, a display and an embedded system to support intelligent sensor data processing and control without the need for external devices such as smartphones. To gain information about the proximate surroundings of the hand, the prostheses embed a camera (OV2640, OmniVision) at the base of the thumb. The camera module has a size of 8 × 8 × 6.3 mm and is connected to the processor's digital camera interface (DCMI) by a 24 pin flat-flex cable. The camera is configured to provide a 176 × 144 pixel RGB image at 10 frames per second. In the female prosthesis, a Time of Flight (ToF) distance sensor (VL53L1X, STMicroelectronics) placed close to the camera is used to measure the distance of a target object to the hand. Relative motor encoders and, in the female version, an IMU (BNO055, Bosch Sensortec) located on the embedded system's PCB provide proprioceptive information. In addition the state of the users forearm can be estimated using the IMU.

**Figure 4:**
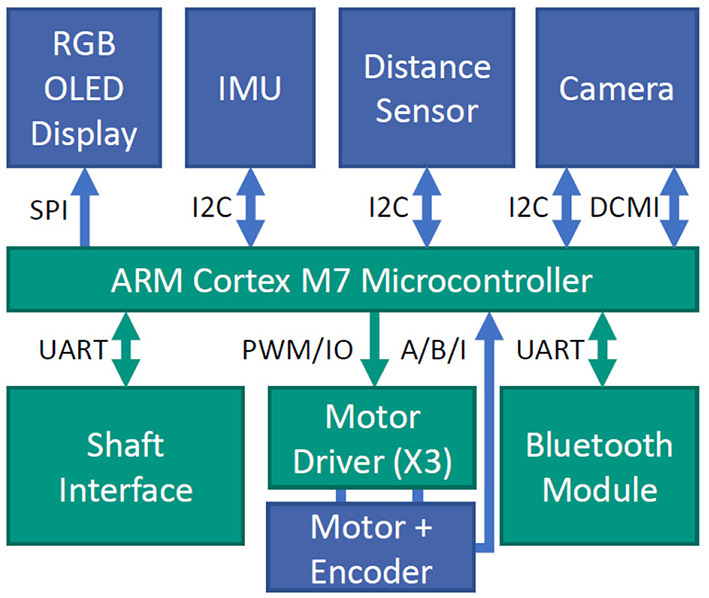
Block diagram showing the functional units of the embedded system. Parts in green are directly placed on the central PCB, the parts in blue are separate components distributed throughout the hand.

For processing the different sensor data and camera images as well as for control, an embedded system is integrated into the hand, directly above the mechanism. An overview of the complete system inside the hand is shown in Figure 4. The system is based on an ARM Cortex-M7 core (STM32H7, STMicroelectronics) running at 400 MHz. The embedded system includes a shaft interface to e.g., connect to a wrist rotation unit or EMG-electrodes.

On the embedded system a resource-aware convolutional neural network is implemented to recognize a set of known objects in the camera image as described in Hundhausen et al. (2019). To this end, an RGB image obtained from the camera is resized to 72 × 72 pixels and is used as input for the network. The hyperparameters of the network architecture are optimized using a genetic algorithm. These hyperparameters include the number of convolution filters, kernel size, strides and pooling types. For more details on the network architecture synthesis we refer the reader to Hundhausen et al. (2019). The cost function used for optimization is rating the network's accuracy after training as well as the network's number of multiply-accumulate-operations during inference. The targeted amount of operations is set to two million operations which allows inference by the given hardware in approximately 150 ms which was identified as an acceptable delay by Farrell and Weir (2007).

The network is trained on a dataset consisting of 13 object classes with 300 images per class. The images are augmented whereby the objects are segmented in the images and the background is replaced by artificially generated noise. For inference, the network is implemented using the CMSIS:NN framework (Lai et al., 2018) that allows optimized execution on the ARM processor. Using this optimized inference, the runtime can be reduced to 23.3 % of the not optimized implementation and inference takes 115 ms. The classification accuracy on the test set of the recorded data amounts to 96.51 %. In addition to the recognition of known objects, we also investigate object segmentation for further estimation of object orientation and for obtaining knowledge about the object shape, see Hundhausen et al. (2021). For this purpose an encoder-decoder network is designed that outputs a pixel-wise mask that segments trained objects in the images. The object class determined by the classification network in combination with inertial sensor data can be used for the selection of a suitable grasp type.

An OLED display in the back of the hand provides feedback to the user about the current status of the hand as well as the proposed grasp type and the orientation for a recognized object.

## 5. Evaluation

The female prosthetic hand is evaluated and compared to the male prosthetic hand to assess the improvement of the design. The evaluation includes the hand characteristics in terms of grasping force, closing speed and hand weight. In addition, an assessment of grasping functionality using an adapted version of the YCB Assessment Protocol is performed and a task-oriented evaluation of object grasping and manipulation is conducted. The context information provided by the multi-modal sensor system is evaluated in a sensor-based grasping experiment.

### 5.1. Prosthesis Characteristics

The grasp force of the prosthesis in a cylindrical power grip is assessed using a sensorized wooden cylinder of 49 mm diameter that integrates a 6D force/torque sensor (Mini 40, ATI Industrial Automation). The cylinder is grasped by the prosthesis with the thumb and the fingers touching on opposite sides of the sensor and held vertically. The individual finger forces are measured by positioning the flat hand directly over the force/torque sensor. By closing the hand, one finger is pressed onto the sensor while the others close freely. This procedure is performed for every finger. Both measurements are repeated 15 times each.

**Figure 5:**
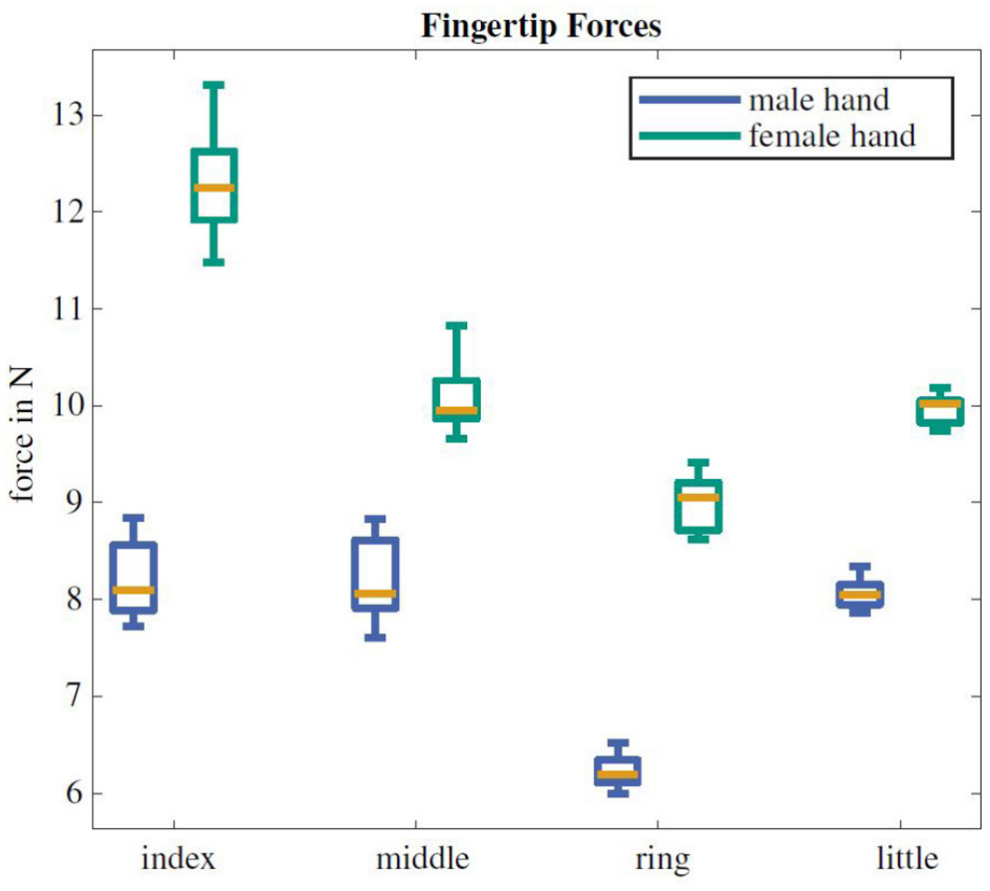
Fingertip forces of the male and female prosthesis. The orange line marks the median force, the box boundaries denote the first and third quartile and the outer lines depict the extrema of the respective fingertip force.

The cylindrical power grasp force amounts to a mean of 24.2 N with a standard deviation of ±1.9 N for the male prosthesis and 40.5 ± 8.1 N for the female hand. The mean finger forces range between 6.2–8.2 N and 9.0–12.3 N for the male and female hands, respectively. The individual forces of the different fingers are shown in Figure 5. The thumb grasp force in an extended configuration amounts to 53.1 ± 1.4 N.

**Figure 6:**
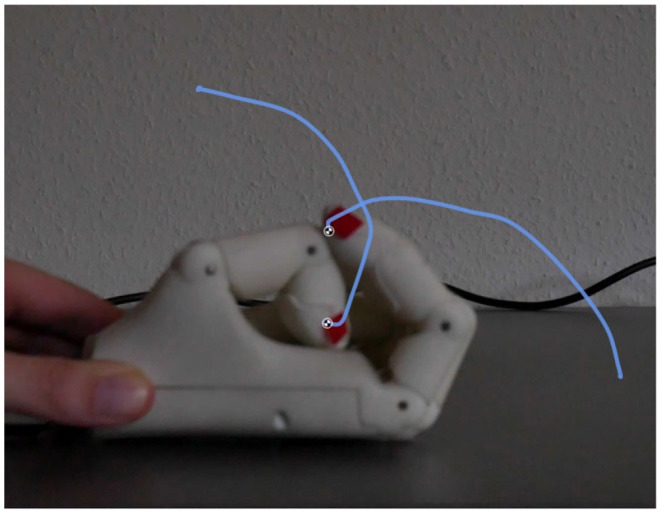
Snapshot from the video evaluation of the female hand closing speed. Red markers on thumb and index finger are tracked in the video sequence, blue lines indicate the closing trajectories of these two fingers.

The hand closing time is measured in an experimental setup, in which we track the fingertip positions of index and thumb in image sequences. To determine the time, we repeated the experiments five times. The hand was placed in front of the camera lying on the back of the hand on a flat surface, exposing thumb and index finger to the camera, see Figure 6. This orientation of the hand represents the worst case for fast closing, as gravity in this orientation pulls the finger open and is hindering fast acceleration of the fingers, whereas rotation of the hand by 180° would result in gravity-assisted finger closing. The finger tips were marked using red tape for color-based tracking. The tracking of one corner of the red tape was performed using the video tracking software *kinovea*^1^ as shown in Figure 6. While the male hand closes entirely in 1.32 s±0.04 s, the female hand exhibits a closing speed of 0.73 s±0.02 s. The nominal maximum motor speed is kept constant for both versions.

**Figure 7:**
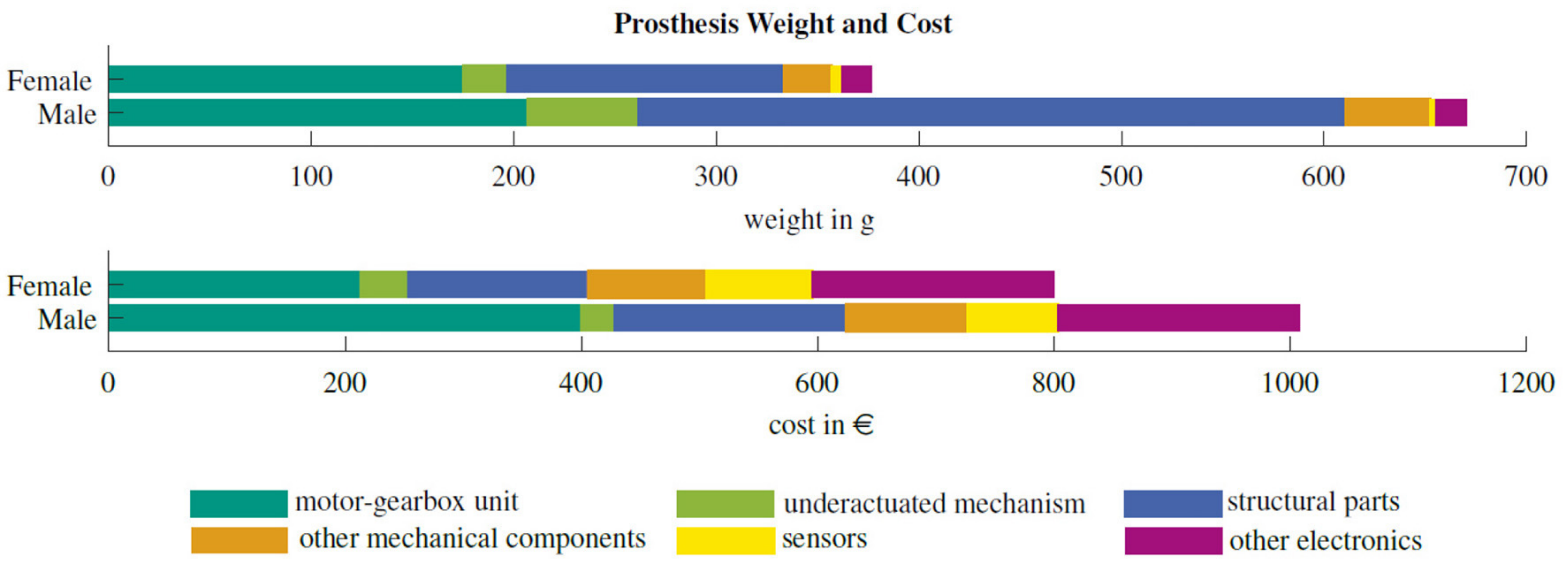
Distribution of weight and cost among the components of the male and female KIT Prosthetic Hand.

The female prosthesis weighs 377 g and requires material costs of 896 €, as shown in Figure 7. The male hand has a weight of 670 g and material costs of 1008 €. The bulk of weight reduction is achieved by optimizing the structural 3D-printed parts for the palm. In contrast to the mechanism in the male version, which was milled from aluminum, the mechanism in the female hand is also 3D-printed, reducing the weight by 60 %. Due to the additional transmission ratio of 2:1 in the mechanism and reduction of the diameter of the motor tendon pulley, additional weight is saved as the motor requires one reduction gear stage less.

### 5.2. Grasping Ability

We evaluated the grasping and manipulation abilities of the hands using 1) the *YCB Gripper Assessment Protocol* to assess grasping abilities and 2) a second *task-oriented protocol* for assessing the hand performance in activities of daily living (ADL).

#### YCB Gripper Assessment Protocol

The general grasping ability is assessed based on the YCB Gripper Assessment Protocol as proposed by Calli et al. (2015). In contrast to the original protocol, we include all object categories from the *YCB Object Set* except for the task items category. This category, containing e.g., a peg-in-hole board or the assembly of an airplane toy, is excluded from the evaluation as we focus on the assessment of the hand grasping abilities. Altogether, 60 objects were tested. No position offsets are applied to the objects as these are compensated by the user. The procedure consists of grasping each object from a table, holding it for 3 s and rotating it by 90°.

The procedure was applied to both the male and female prosthetic hand while being manually controlled by a human operator. One point is scored if the object is successfully lifted and held. A second point is scored if the object does not move or slide inside the hand, a third point is scored if the object remains grasped after the rotation and the fourth point is scored if the object does not move inside the hand after rotation. The maximum score that can be achieved for each object is four. For the articulated objects (table cloth, chain, rope, t-shirt) the object is grasped and lifted three times and half a point is granted for each successful attempt.

The scores were 193 and 203.5 of the possible 230 points for the male and female hand, respectively. In total 85.2 % of all objects could be grasped with the male hand and 91.8 % with the female hand. Both hands encounter difficulties in grasping thin and small objects like credit cards, nails and washers. Despite the smaller size of the female hand, there are no notable shortcomings in grasping large objects, like the wood block or the mini soccer ball from the YCB Object Set. Both hands are able to lift the heavy objects from the YCB object with a full score, for example the power drill, the table cloth and the wood block. The skillet could be lifted at the handle, but moved inside the hand during hand rotation due to the high torque on the handle.

#### Task-Oriented Protocol

**Figure 8:**
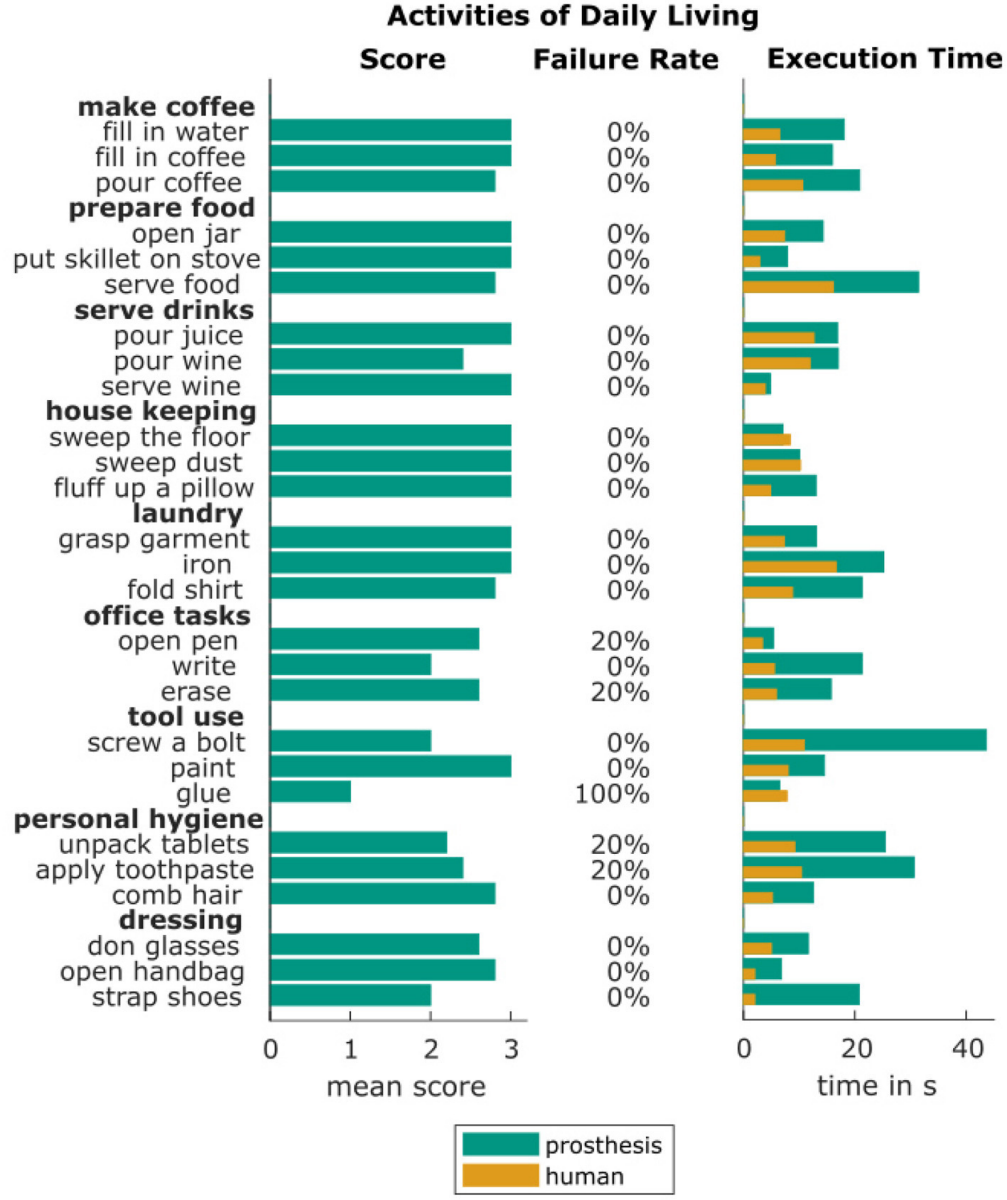
Tasks performed in the task-oriented protocol with the mean prosthesis scores, ranging between 0 for the hand being unable to grasp the object to 3 for a comfortable task execution, the rate of failed task executions over five trials and the mean execution times with the prosthetic hand and an able human hand.

The female hand is additionally evaluated with a task oriented protocol of common daily life activities. To this end, the prosthesis was mounted on a shaft, which can be worn below the forearm of the able hand and several activities of daily living were performed using the prosthesis. The tasks are selected based on the objects and activities proposed by Matheus and Dollar (2010). The list of the tasks is shown in Figure 8.

The execution of every task is repeated five times. The task execution quality is assessed with a score between 0 and 3 points. The used scoring system is designed as follows: one point is granted for achieving a stable grasp, a second point is granted for successful accomplishment of the task goal and the third point is granted when the task execution is done in a natural and comfortable manner compared to its execution with two able human hands. As an example, for the writing task, the first point is scored if the pen is stably held in the hand, the second point for writing the requested sentence on a piece of paper in a readable manner and the third point is granted only if the handwriting looks natural, the task is executed in a comfortable manner and the writing time is not disproportionally long. As defined in the Southampton Hand Assessment Procedure (SHAP) by Light et al. (2002), each task needs to be solved within eight times the time needed by an able-bodied person to be not considered disproportionally long. If the task execution requires more time, it can only be rated with two points at maximum.

The scores and execution times achieved with the female prosthesis are shown in Figure 8. In addition, the task failure rate over all five executions of each task is given. Over all activities, the task was not fulfilled successfully in 6.7 % of all executions. The overall score of 88.6 % of achievable points indicates a satisfying functionality of the hand in performing activities of daily life.

The prosthesis was especially successful in executing everyday household activities like food preparation, house keeping and laundry. Lower task evaluation scores are mainly seen in office tasks as well as medicating and bathing tasks. This is due to the fact, that these tasks require more complex grasping and prehensile in-hand object manipulation. The only task that could not be accomplished by the prosthesis was gluing with a hot glue gun. While the gun could be grasped, the trigger could not be pressed by the index finger. The task of screwing a bolt into a nut was especially challenging, since the hand is not able to turn the screw driver within the hand, but instead the full hand needs to be rotated with the screwdriver. This results in unnatural and uncomfortable whole-body compensatory movements. No task took more than eight times the time of an execution with two able human hands. Strapping a shoe was the only task that exceeded the defined time constraint because the task took 10.4 times the time needed by a human with two able hands.

### 5.3. Sensor-Based Grasping

**Figure 9:**
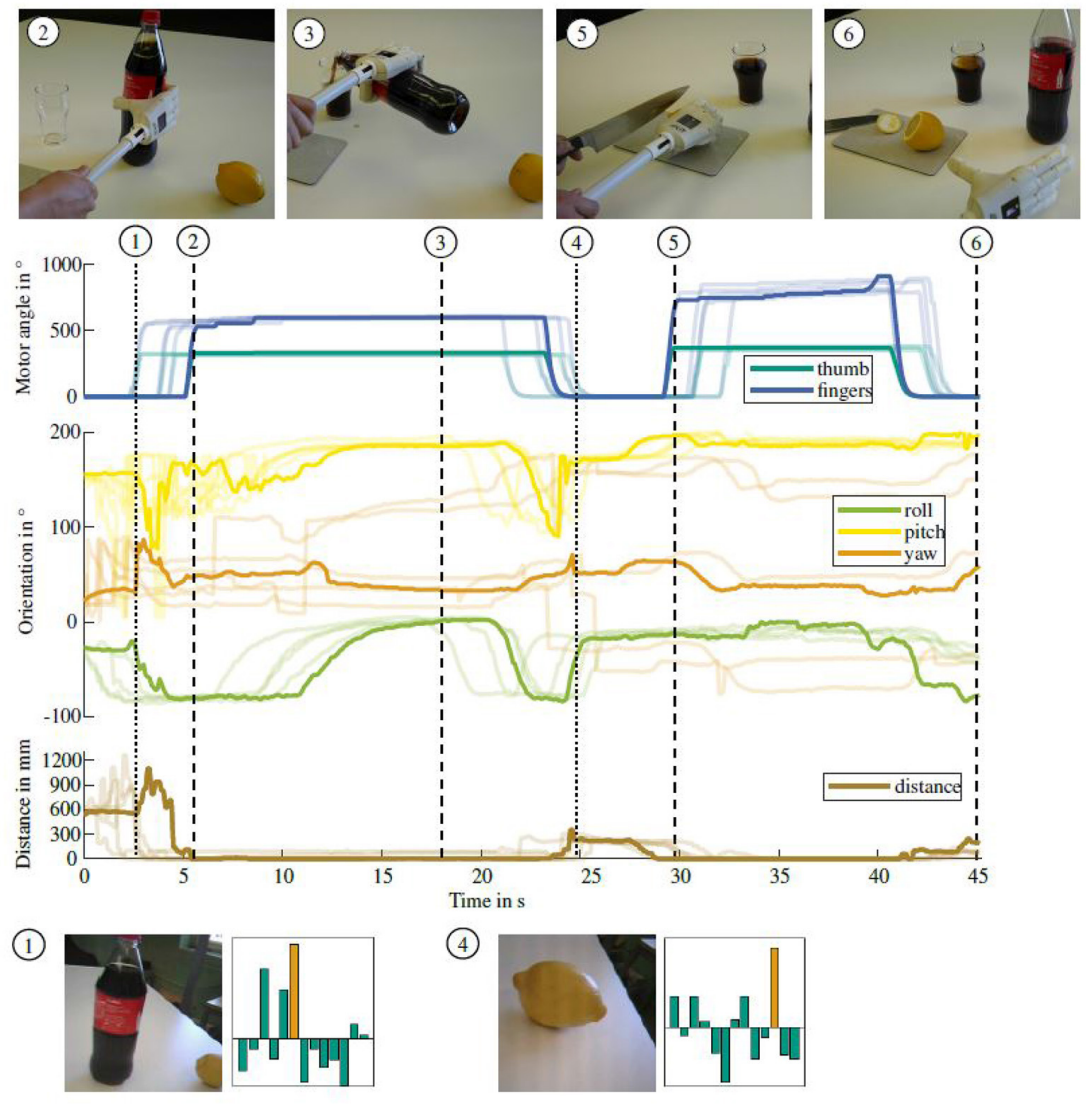
Sensor readings while pouring coke into a glass and adding a slice cut off from a lemon. Graphs show an exemplary measurement of the motor positions, hand orientation from the IMU and object distance. Four additional experiments printed in the background underline the reliability of the sensor data. Important events of the grasping process are marked by dashed lines and corresponding images of the scene are shown above the graphs. The triggering of the object recognition is marked by dotted lines and an images captured by the hand camera together with the object recognition probabilities are shown below the graphs. The recognition probability of the coke bottle and lemon, respectively are marked in orange in the bar chart, indicating the object was recognized correctly.

The merit of the multi-modal sensor system for grasp control is evaluated in the context of sensor-based grasping. All sensor readings are recorded and evaluated during two different grasping sequences of daily living activities. In the first sequence, a bottle of coke is grasped with the prosthesis, opened and the coke is poured into a glass. After the bottle is placed back on the table, a lemon is grasped and held firmly. A slice is cut off with a knife in the second hand and the lemon is placed on the table. The lemon slice is inserted into the glass of coke with the able hand. Before grasping, an image of the object is captured by the hand's integrated camera and the object recognition is run on the in-hand integrated embedded system. Figure 9 shows the experimental procedure, the sensor readings and results of the object recognition. The camera image for object recognition is shown together with the recognition probabilities for all 13 trained objects in the bottom row. The correct object, being coke and lemon, respectively, is marked in orange in the bar chart diagrams. In both cases, the object recognition returns the highest probability for the correct object, allowing for object-specific grasp control. An in-depth evaluation and discussion of the object recognition algorithm can be found in our previous work in Hundhausen et al. (2019).

The sensor readings for five executions of the task are shown in the middle of Figure 9. The associated sensor readings are plotted in solid lines for an exemplary execution and in transparent lines for the remaining four executions. All sensor readings have been normalized over the execution time, to show the similarity of the acquired sensor data throughout several executions. Grasping the bottle is finished after 5.2 s, which is clearly visible in the motor position data. Similarly the bottle is placed on the table after 23.3 s, coincident with the motor position moving back to the initial state. Grasping and releasing the lemon occur at 29.9 s and 40.6 s, respectively.

Approaching the object can also be inferred from the distance sensor in the palm, which shows a decrease of the object distance from 379 mm to 15 mm between 3.6 s and 5.1 s. The grasping action can therefore be controlled based on the distance to the object which is provided by the distance sensor. As the ball of the thumb does not touch the bottle, the distance sensor does not decrease to zero throughout the grasp. The release of the object, which is also visible in the finger motor positions, is consequently followed by an increase of the object distance starting after 24.6 s.

The orientation data from the IMU provides additional information about the grasp. Figure 9 shows the hand orientation in the hand coordinate system. Several rotations of the prosthesis throughout the manipulation action can be recognized. The recording starts with the hand in a horizontal position and the palm facing toward the table. After 11.1 s, when the bottle is grasped and opened, the prosthesis starts rotating with the bottle to pour coke into the glass. This is visible in the roll angle of the IMU. Once the pouring action is finished and the hand is rotated back, the placement of the bottle can be recognized based on the distance sensor data. The disturbance induced by opening the bottle and placing it back on the table can be seen in the hand's pitch angle.

**Figure 10:**
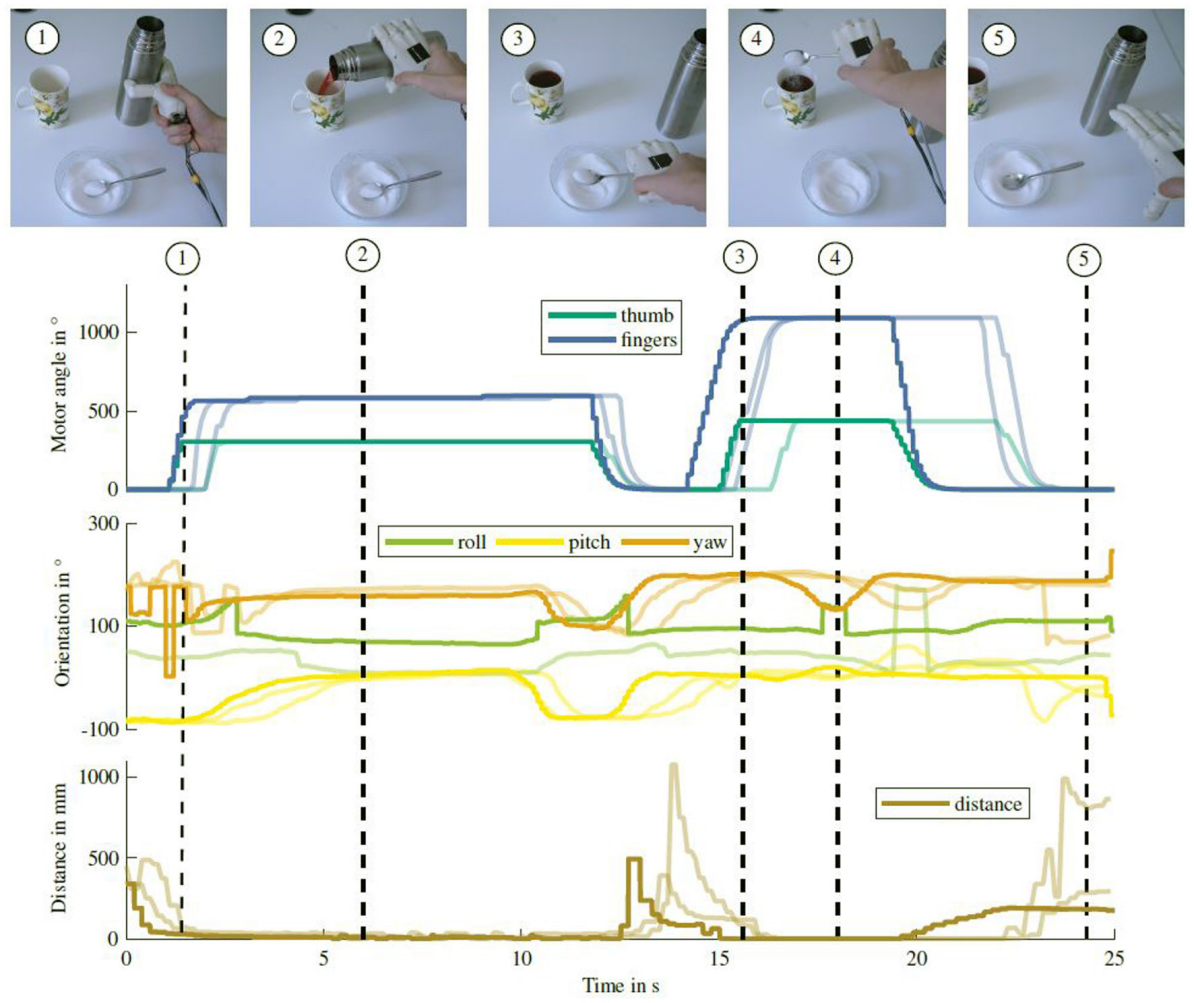
Sensor readings while pouring tea into a cup and adding sugar with a spoon. Graph notation is similar to the lemonade preparation task shown in Figure 9.

To grasp the lemon, the hand is again horizontally orientated, as visible in the roll angle of the IMU. An additional example of pouring tea into a cup and adding sugar with a spoon is shown in Figure 10. Similar to the lemonade preparation task, different events throughout the task can be recognized based on the sensor readings. The grasps can be seen in an increase in motor position and a decrease in object distance. The tea pouring as well as tilting the spoon to add the sugar can be recognized in the IMU orientation. Both experiments show that information about the current phase of an object manipulation task can be inferred from sensor data and can be used for semi-autonomous grasp control.

## 6. Discussion and Conclusion

We present the KIT Prosthetic Hands as an example for intelligent prostheses equipped with abilities needed for the realization of semi-autonomous grasping. The hands are designed to support users in grasping objects to master daily life activities. The intelligence of the hands is achieved by combining adaptive underactuated mechanisms with a multi-modal sensor system and a resource-aware embedded system for onboard processing of sensory information and control. Thanks to the underactuated mechanism, high grasp forces can be achieved. The on-board processing of visual information relevant to the current task allows the implementation of semi-autonomous grasping behaviors.

The hand's size and weight comply to the requirements for a hand prosthesis. With its total weight of 377 g, the hand is lighter than any commercial myoelectric prosthetic hand as presented in Table 1, and is comparable to the human hand with approximately 400 g (Kaye and Konz, 1986). Compared to the male hand, the female hand shows a reduction of 44 % in weight and 30 % in cost. As shown in Figure 7, this is achieved by a significant improvement in lightweight design of mechanism and structural hand parts as well as the 3D-printed design of the mechanism without custom metal parts. Compared to the male hand, the closing time of the female prosthesis is decreased by 0.59 s to an absolute closing time of 0.73 s. This increase in speed is achieved by the improved mechanism design and the shorter finger dimensions requiring a smaller tendon deflection. The hand provides a cylindrical grasp force of 40.1 N and a mean fingertip force of 10.3 N within the four fingers. Compared to the male hand, the increase of the finger forces amounts on average to 35.2 %. This is within the range of commercial and research prosthetic hands as e.g., the iLimb Pulse (Belter and Dollar, 2013) or the SSSA-MyHand by Controzzi et al. (2017). With 53.4 N, the thumb is capable of providing a significantly higher force to counteract the four fingers.

The evaluation of the prosthesis based on the YCB Gripper Assessment Protocol shows a grasp functionality of 91.8 % in grasping everyday objects and the prosthesis achieves a score of 88.6 % in the execution of daily activity manipulation tasks. This shows the potential of the hand to support users throughout their daily life spanning food preparation, household and hygiene tasks, but also including their professional life, exemplary shown in office and workshop activities. The improvements of the female prosthetic hand over the male version are summarized in Table 3.

**Table 3:** Key characteristics of the male and female KIT prosthetic hands.

**Prosthesis**	**Percentile**	**Weight**	**Material**	**Embedded**	**Grasping**	**Closing**	**YCB GAP**
			**cost**	**sensors**	**force**	**speed**	**score**
Male	50th male	768 g	1,008€	Camera,	24.2 N ± 1.9 N	1.32 s ± 0.04 s	193
Female	50th female	377 g	896€	Distance IMU, Camera	40.5 N ± 8.1 N	0.73 s ± 0.02 s	203.5

With these achievements, we provide important prerequisites for novel generation of prosthetic hands that integrate multi-modal sensing and resource-aware computing for the realization of semi-autonomous grasping and improving the way how users can interact with their prosthetic hands in an easy and intuitive way. We believe that the hardware design of the KIT Prosthetic Handas an intelligent and functional hand prosthesis provides a powerful platform for the development of intelligent, semi-autonomous control algorithms.

In the future we plan to design and implement a semi-autonomous control scheme that makes use of the multi-modal sensor data and the embedded system. Our goal is to endow the prosthesis with functionalities for semi-automatic preshape selection based on object recognition and IMU data as well as grasp execution based on distance information. All control algorithms and sensor data processing will be performed on the embedded system, eliminating the need for external sensor and processing resources.

**Figure 11:**
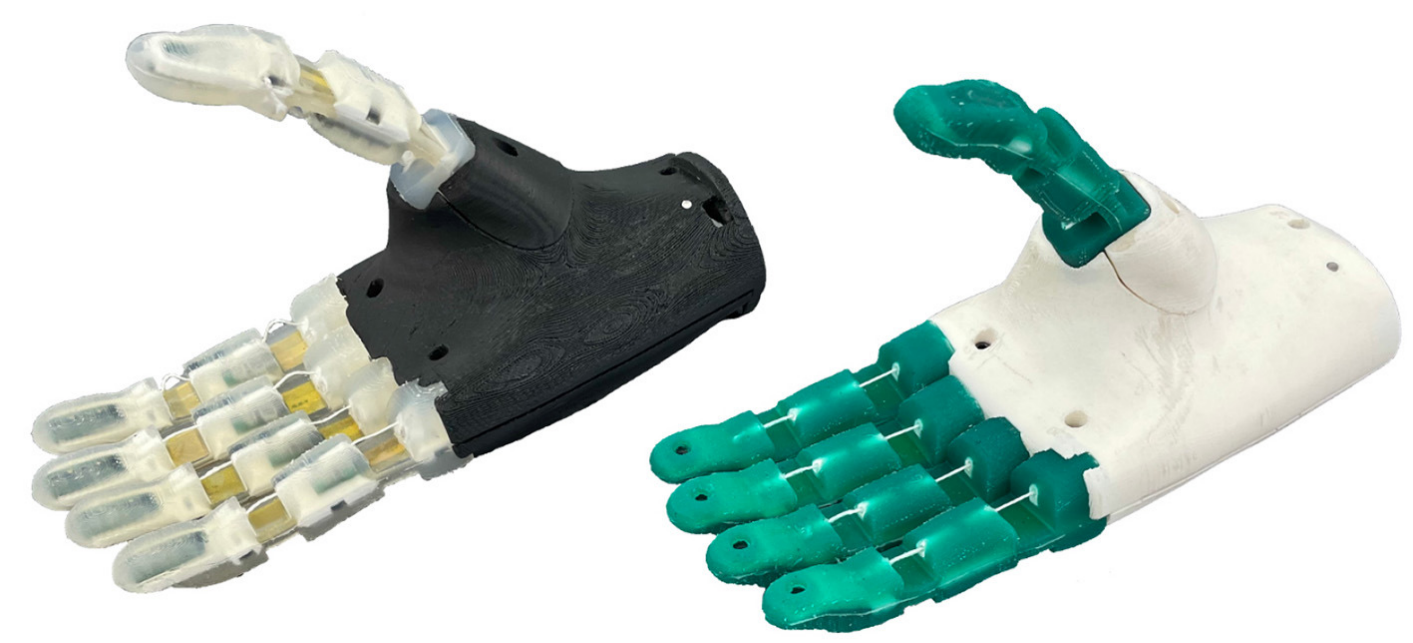
The KIT sensorized soft hand (left) and KIT finger-vision soft hand (right) inspired by the prosthetic hand development.

Important to mention is also the fact that the underactuated mechanisms used in the KIT prosthetic hands served as the basis for the development of the hands of the humanoid robot ARMAR-6 by Asfour et al. (2019). In addition, the new version of prosthetic hand, the female version, served as a basis for the development of several new soft humanoid robotic hands, the KIT Finger-Vision Soft Hand, see Hundhausen et al. (2020), and the KIT Sensorized Soft Hand with tactile sensing of the fingers, see Weiner et al. (2021). Both hands are shown in Figure 11. The hands allow an individual actuation of the thumb and the index finger. Both are driven by three motors and include an adapted version of the underactuated mechanism described in Section 4, that is designed to drive only three fingers with the same motor. The robotic hands are equipped with soft, monolithic fingers with joints and phalanx bodies made from silicone and a rigid skeleton structure in each finger phalanx. The fingers are actuated by tendons routed through the silicone. A flat band of non-stretchable material in the neutral axis of the finger provides a spring-like behavior to enhance the finger bending trajectory and protects cables routed to the sensors within the fingers. The soft robotic hands are equipped with different sensor setups in the fingers. The KIT Finger-Vision Soft Hand features one camera in the tip of each finger, as described in Hundhausen et al. (2020). The fingers of the KIT Sensorized Soft Hand are equipped with a multi-modal haptic sensor system Weiner et al. (2021). In both hands, the raw sensor information is processed in-hand on a high-performant embedded system based on the same ARM Cortex-M7 core that is also used for the prosthetic hand presented in this work. In addition, an FPGA (Artix 7, Xilinx) is integrated to enable hardware-accelerated processing of the high amount of incoming sensory data. The soft robotic hands have a length of 215 mm and a weight of about 580 g.

## Data Availability Statement

The original contributions presented in the study are included in the article/supplementary material, further inquiries can be directed to the corresponding authors.

## Author Contributions

SR designed the mechanics of the prosthetic hand with JS. The concept of the underactuated mechanism is based on the work of TA and was adapted by PW. PW and FH designed the sensor setup and embedded system of the prosthesis. FH, PW, and JS developed the firmware of the embedded system. The experiments were designed and conducted by JS and PW. The entire work was conceptualized and supervised by TA. The manuscript was written jointly by all authors.

## Funding

This work has been supported by the German Federal Ministry of Education and Research (BMBF) under the project INOPRO (16SV7665) and by the Carl Zeiss Foundation through the JuBot project.

## Conflict of Interest

The authors declare that the research was conducted in the absence of any commercial or financial relationships that could be construed as a potential conflict of interest.

## Publisher's Note

All claims expressed in this article are solely those of the authors and do not necessarily represent those of their affiliated organizations, or those of the publisher, the editors and the reviewers. Any product that may be evaluated in this article, or claim that may be made by its manufacturer, is not guaranteed or endorsed by the publisher.

## Footnotes

^1^ https://www.kinovea.org/
